# The Role of Satellite DNAs in Genome Architecture and Sex Chromosome Evolution in Crambidae Moths

**DOI:** 10.3389/fgene.2021.661417

**Published:** 2021-03-30

**Authors:** Diogo C. Cabral-de-Mello, Magda Zrzavá, Svatava Kubíčková, Pedro Rendón, František Marec

**Affiliations:** ^1^Departamento de Biologia Geral e Aplicada, Instituto de Biociências/IB, UNESP—Univ Estadual Paulista, Rio Claro, Brazil; ^2^Biology Centre, Czech Academy of Sciences, Institute of Entomology, České Budějovice, Czechia; ^3^Faculty of Science, University of South Bohemia, České Budějovice, Czechia; ^4^Veterinary Research Institute, Brno, Czechia; ^5^IAEA-TCLA—Consultant—USDA-APHIS-Moscamed Program Guatemala, Guatemala City, Guatemala

**Keywords:** Lepidoptera, repetitive DNAs, holocentric chromosomes, tandem repeat, W chromatin

## Abstract

Tandem repeats are important parts of eukaryotic genomes being crucial e.g., for centromere and telomere function and chromatin modulation. In Lepidoptera, knowledge of tandem repeats is very limited despite the growing number of sequenced genomes. Here we introduce seven new satellite DNAs (satDNAs), which more than doubles the number of currently known lepidopteran satDNAs. The satDNAs were identified in genomes of three species of Crambidae moths, namely *Ostrinia nubilalis*, *Cydalima perspectalis*, and *Diatraea postlineella*, using graph-based computational pipeline RepeatExplorer. These repeats varied in their abundance and showed high variability within and between species, although some degree of conservation was noted. The satDNAs showed a scattered distribution, often on both autosomes and sex chromosomes, with the exception of both satellites in *D. postlineella*, in which the satDNAs were located at a single autosomal locus. Three satDNAs were abundant on the W chromosomes of *O. nubilalis* and *C. perspectalis*, thus contributing to their differentiation from the Z chromosomes. To provide background for the *in situ* localization of the satDNAs, we performed a detailed cytogenetic analysis of the karyotypes of all three species. This comparative analysis revealed differences in chromosome number, number and location of rDNA clusters, and molecular differentiation of sex chromosomes.

## Introduction

Sex chromosomes are extremely dynamic components of the genomes with high inter- and intraspecific variability. They evolved *de novo* multiple times from an ordinary pair of autosomes across various Eukaryote taxa (Charlesworth et al., 2005; Wei and Barbash, 2015; Abbott et al., 2017; Furman et al., 2020) *via* acquisition of a master sex-determining locus in one of the two homologs of an autosomal pair (Bull, 1983; Charlesworth, 1991; Wright et al., 2016; Furman et al., 2020). This event is followed by suppression of recombination, which leads to accumulation of repetitive DNAs and gene pseudogenization. Ultimately, this gradual genetic erosion may result in the disappearance of Y or W chromosomes (Wei and Barbash, 2015; Abbott et al., 2017).

Accumulation of repeats leads to heterochromatinization of Y or W in many species (Wei and Barbash, 2015; Abbott et al., 2017). Repetitive DNAs that accumulate on sex chromosomes often include satellite DNAs (satDNAs). These non-coding sequences occur in the genomes in hundreds to thousands of copies arranged in tandem in a head-to-tail manner. In general, satDNAs are highly dynamic and their nucleotide sequence, copy number, length of monomers, and chromosomal location can change quickly (Garrido-Ramos, 2017). Empirical data obtained from various animal taxa, such as orthopterans (Palacios-Gimenez et al., 2017; Ferretti et al., 2020), mammals (Acosta et al., 2007; Escudeiro et al., 2019), fishes (Crepaldi and Parise-Maltempi, 2020; Serrano-Freitas et al., 2020), reptiles (Giovannotti et al., 2018), and anurans (Gatto et al., 2018), have demonstrated the role of satDNAs in sex chromosome differentiation.

Lepidoptera (moths and butterflies) with about 160,000 described species (Van Nieukerken et al., 2011) is the largest animal group with female heterogamety (Traut et al., 2007). Most lepidopterans harbor a simple WZ sex chromosome system in females, although Z0 or multiple sex chromosomes have been reported in some species (Traut et al., 2007; Šíchová et al., 2015; Hejníčková et al., 2019). Extensive efforts to understand the sex chromosome composition and evolution in Lepidoptera have been made using variable approaches, from classical cytogenetics to Z-linked gene mapping, comparative genomic hybridization (CGH) of male and female genomic DNAs (gDNAs), genomic *in situ* hybridization (GISH) of female gDNA, W-chromosome painting, and sequencing of male and female genomes (Abe et al., 2005; Yoshido et al., 2005, 2020; Traut et al., 2007, 2013; Vítková et al., 2007; Nguyen et al., 2013; Šíchová et al., 2013; Fraïsse et al., 2017; Zrzavá et al., 2018). These studies documented that the Z is a gene-rich, autosome-like chromosome with conserved synteny (Van’t Hof et al., 2013; Dalíková et al., 2017a). In contrast, the W chromosome is largely heterochromatic, composed of repetitive sequences and mostly lacking protein-coding genes (Traut et al., 2007; Sahara et al., 2012). The heterochromatin nature of the W chromosomes leads to the formation of sex chromatin, a roundish body in interphase nuclei of females (Smith, 1945; Traut and Marec, 1997).

Due to the prevalence of repeats, attempts to sequence the lepidopteran W chromosome are scarce (Abe et al., 2005; Fuková et al., 2007; Traut et al., 2013). These efforts, however, have shown that the W consists mainly of mobile elements. Other studies that focused directly on satDNA detected a few satellites located on the W chromosome in several species, namely in *Plodia interpunctella* (Dalíková et al., 2017b), *Mamestra brassicae* (Mandrioli et al., 2003), and *Spodoptera frugiperda* (Lu et al., 1994). However, low amounts of satDNA appear to be a general feature of lepidopteran genomes, which are generally rather small, ranging from 0.29 pg in *Danaus plexippus* (Danaidae) to 1.94 pg in *Euchlaena irraria* (Geometridae) (Gregory, 2020, Animal Genome Size Database). So far, only a total of five satDNAs have been identified in all lepidopteran species investigated (Lu et al., 1994; Mandrioli et al., 2003; Mahendran et al., 2006; Věchtová et al., 2016; Dalíková et al., 2017b). They showed variable patterns of chromosomal distribution including W specific satDNA (Dalíková et al., 2017b), satDNA shared exclusively by Z and W chromosomes (Mandrioli et al., 2003), and satDNAs spread on multiple chromosomes (Mahendran et al., 2006; Věchtová et al., 2016).

The small sizes of lepidopteran genomes and their repetitive composition, mainly consisting of transposable elements (TEs), may have hindered the isolation of satDNAs using classical methods, i.e., restriction endonuclease digestion of gDNA, which depends on high abundance of a particular repeat (Camacho et al., 2015). Only recently, this difficulty has been overcome by bioinformatic analysis of low-coverage sequenced genomes using RepeatExplorer (Novák et al., 2013). This approach allowed the characterization of multiple satDNAs and generated valuable chromosomal and genomic information, for example in crickets (Palacios-Gimenez et al., 2017), *Drosophila* species (Silva et al., 2019), fishes (Silva et al., 2017; Crepaldi and Parise-Maltempi, 2020), amphibians (da Silva et al., 2020), and some plants (Mata-Sucre et al., 2020; Zwyrtková et al., 2020). However, this approach has not yet been applied to Lepidoptera.

The lepidopteran family Crambidae has more than 10,000 species described worldwide (Nuss et al., 2003–2020), some of which are serious pest of agricultural crops such as sugarcane, maize, rice, and sorghum, and are economically important (Solis, 1997; Munroe and Solis, 1999; Meissle et al., 2010). Despite the overall significance of crambid moths, very little is known about their genome architecture, because cytogenetics of the Crambidae was poorly explored. In a few crambid species, only chromosome numbers are known (Robinson, 1971), including the reduced *n* = 17 in the sugarcane borer *Diatraea saccharalis* (Virkki, 1963) and the ancestral *n* = 31 in *Ostrinia* representatives (Guthrie et al., 1965; Kageyma and Traut, 2004; Yasukochi et al., 2016). Sex chromosomes have only been studied in *O. scapulalis* (Kageyma and Traut, 2004) and *O. nubilalis* (Yasukochi et al., 2016). In *O. nubilalis*, a more detailed chromosomal analysis was performed with the assignment of the 31 chromosomes by gene-based fluorescence *in situ* hybridization (FISH) mapping (Yasukochi et al., 2016). To expand our knowledge of this group, we analyzed karyotypes and genomes of three representatives of Crambidae, namely the European corn borer (*Ostrinia nubilalis*), one of the most important pests causing economical losses to corn growers (Lynch, 1980), the box tree moth (*Cydalima perspectalis*), an Asian species that has been introduced in Europe, causing defoliation of the ornamental shrub *Buxus* spp. (Nacambo et al., 2013), and the Guatemalan sugarcane borer (*Diatraea postlineella*), a pest which causes significant damage to sugarcane in Guatemala (Solis and Metz, 2016; Solis et al., accepted). Our aim was to investigate the role of tandem repeats in general architecture of genomes and in sex chromosome differentiation in studied species, as little is known about these sequences in Lepidoptera, which are otherwise important components of eukaryotic genomes. To accomplish this, we used for the first time in Lepidoptera clustering analysis performed by RepeatExplorer on male and female genomic sequences of all three species to identify and map satDNAs. Further, we characterized the chromosomes of the studied species in order to provide a cytogenetic background not only to our FISH experiments but also to existing or upcoming sequencing projects. In particular, we focused on sex chromosomes, knowledge of which is important for potential pest control, such as the sterile insect technique, as *O. nubilalis* is one of the most important pests causing economical losses to corn growers (Lynch, 1980).

## Materials and Methods

### Animals

We studied three species of the family Crambidae, the box tree moth (*C. perspectalis*), the Guatemalan sugarcane borer (*D. postlineella*), and the European corn borer (*O. nubilalis*). A small laboratory colony of *C. perspectalis* was established from the last instar larvae collected from infested shrubs of *Buxus* spp. in Valtice (Czechia) in May 2018. The colony was kept for several generations on fresh leaves of *Buxus* spp. Eggs of *D. postlineella* were obtained from a mass-reared colony at the Santa Ana sugarcane farm, Escuintla, Guatemala City, Guatemala. Larvae of this species were reared on artificial diet prepared according to the supplier’s recipe. *O. nubilalis* (Z and E strains) was obtained from a laboratory colony kept at the Department of Entomology, Max Planck Institute of Chemical Ecology, Jena, Germany. For genomic analysis, we used sequenced genomes from both *O. nubilalis* strains, but only Z-strain was used for further chromosomal studies. Larvae were reared on an artificial wheat germ-based diet (Lewis and Lynch, 1969) with some modifications, and adults fed a 10% solution of honey in water. Cultures of all three species were maintained at 20–22°C and 12-h light/12-h dark regime.

### Polyploid Nuclei Preparation and Microdissection of the W Chromatin

Polyploid interphase nuclei were prepared from Malpighian tubules of male and female larvae of the last instar (see Marec and Traut, 1994). Malpighian tubules were dissected in physiological solution, fixed in Carnoy’s fixative (ethanol, chloroform, acetic acid, 6:3:1) on a slide for about 1 min, stained with 1.25% lactic acetic orcein for 3–5 min, and mounted in the staining solution. The slides were then examined under a light microscope for the presence of W chromatin in females and its absence in males (reviewed in Traut and Marec, 1996).

For laser microdissection of W chromatin bodies, we followed the procedure described in Fuková et al. (2007) with some modifications. Malpighian tubules were dissected from last instar female larvae, swollen for 10 min in a hypotonic solution (75 mM KCl) and fixed in Carnoy’s fixative for 15 min. The tubules were then transferred into a drop of 60% acetic acid on a glass slide coated with a polyethylene naphthalate membrane (Goodfellow, Huntingdon, United Kingdom) and torn to pieces using tungsten needles. The cell suspension was spread at 40°C using a heating plate and stained with 5% Giemsa for 7 min. Microdissection of W chromatin bodies was performed using the PALM MicroLaser System (Carl Zeiss MicroImaging GmbH, Munich, Germany) as described in Kubickova et al. (2002).

### Chromosome Preparations

Spread chromosome preparations were made as described previously (Mediouni et al., 2004; Šíchová et al., 2013). Mitotic chromosomes were obtained from wing imaginal disks of the last instar larvae of both sexes and meiotic chromosomes in the pachytene stage from testes of the penultimate and last instar larvae and ovaries of the last instar larvae and early pupae. Wing imaginal disks and testes were dissected in a physiological solution, hypotonized for 10 min in 75 mM KCl and fixed in Carnoy’s solution for at least 15 min, whereas the ovaries were transferred to Carnoy’s solution for 15 min immediately after dissection. The fixed tissues were macerated on a slide in a drop of 60% acetic acid and then spread at 45°C using a heating plate. The slides were inspected under a phase contrast microscope and preparations of sufficient quality were passed through an ethanol series (70, 80, and 100%, 30 s each) and stored at –20°C until use.

### Genome Sequencing, Satellite DNA Identification and Analysis

Genomic DNA was extracted from one male larva and one female larva of each species using the CTAB (hexadecyltrimethylammonium bromide) DNA isolation procedure according to Winnepenninckx et al. (1993) with the following modifications. The concentration of EDTA in the extraction buffer was doubled, i.e., 40 mM, the homogenized material was incubated overnight, the centrifugation steps were performed at 14,000 *g* and prior to isopropanol precipitation, the samples were treated with 62.25 μg/mL RNase A for 30 min at 37°C.

Paired-end sequencing (2 × 150 bp) using the Illumina HiSeq 4000 system was performed by Novogene (HK) Co., Ltd., (Hong Kong, China). We checked the quality of reads with FastQC (Andrews, 2010) and processed the reads though Toolkit suit using default options (Gordon and Hannon, 2010) to be used as input for RepeatExplorer (available at^1^) clustering. We made separate identification of satDNAs for each three species. A comparative analysis of satDNAs between the sexes for *D. postlineella* and *C. perspectalis* was performed using 250,000 reads of each sex as input. For *O. nubilalis*, genomes of both sexes from the two strains (E and Z) were used, being 125,000 reads per genome. After clustering by RepeatExplorer (Novák et al., 2010, 2013) using default options, the satDNAs identified as satDNAs by TAREAN tool (Novák et al., 2017) were considered for subsequent analysis. The tandem arrangement was checked by dotplot and Tandem Repeats Finder, TRF (Benson, 1999).

For similarity analysis, we performed all-against-all comparison of monomers of the recovered satDNAs using RepeatMasker (Smit et al., 2013–2015) and the “rm_homology.py” script^2^. We used RepeatMasker to calculate divergence and abundance of each satDNA in the female and the male genomes at intraspecific level. For this purpose, we randomly selected 7.5 million read pairs per library obtained by seqtk tool^3^ and aligned them against dimers of consensus satDNA sequences. To estimate the average Kimura 2-parameter distances (K2P) for each satDNA family, we used the calcDivergenceFromAlign.pl script from the RepeatMasker utility tool. Genomic abundance for each satDNA family was calculated according to the proportion of nucleotides aligned with the reference consensus sequence divided by the library size. Finally, we compared the divergence of satDNAs between sexes generating repeat landscapes showing the relative abundance of repeat elements on the Y-axis and 1% intervals of K2P distance from the consensus on the X-axis. In addition, we also performed the RepeatMasker analysis at interspecific level to check the occurrence of different satDNAs in the species studied and to check their abundance. Similarity of the satDNAs identified with previously characterized sequences was checked by blast search in NCBI and Repbase.

### DNA Probes and Fluorescence *in situ* Hybridization

For each studied species, we prepared specific gDNA probes, W-chromosome painting probes, and satDNA probes. We also prepared non-species specific probes to localize conserved sequences including telomeric repeats and 18S rDNA. These probes were used for different FISH experiments following different protocols, as specified below.

Female and male gDNAs extracted by the CTAB procedure were labeled by nick translation with Cy3-dUTP for female gDNA or fluorescein-12-dUTP (both Jena Bioscience, Jena, Germany) for male gDNA, or vice versa. The nick translation mixture was composed of 500 ng of gDNA, 25 μM of each dATP, dCTP, and dGTP, 9 μM of dTTP, 16 μM of labeled nucleotides, nick translation buffer (50 mM Tris–HCl, pH 7.5, 5 mM MgCl_2_, 0.005% BSA), 10 mM β-mercaptoethanol, 20 U DNA Polymerase I (ThermoFisher Scientific, Waltham, MA, United States), and 0.005 U DNase I (ThermoFisher Scientific). The mixture was incubated for 2 h at 15°C. These labeled gDNA probes were used for CGH following the protocol described in Traut et al. (1999) with modifications proposed by Dalíková et al. (2017a).

W-chromosome painting probes were prepared as described in Fuková et al. (2007). Briefly, samples of microdissected W chromatin were amplified by PCR using a GenomePlex Single Cell Whole Genome Amplification Kit, WGA4 (Sigma-Aldrich, St. Louis, MO, United States). The amplified product was re-amplified and labeled with either Cy3-dUTP or fluorescein-12-dUTP by PCR using a GenomePlex WGA Reamplification Kit, WGA3 (Sigma-Aldrich). These probes were used in W-chromosome painting experiments at intraspecific level and for cross-species W-painting following the CGH protocol (Traut et al., 1999). For intraspecific experiments, we combined the W-painting probe with a (TTAGG)*_*n*_* telomeric probe obtained by non-template PCR according to Ijdo et al. (1991) and using primers (TTAGG)*_5_* and (CCTAA)*_5_*. The telomeric probe was labeled with Cy3-dUTP by using the improved nick translation procedure of Kato et al. (2006) with some modifications (see Dalíková et al., 2017a).

18S rDNA and satDNA probes were amplified by PCR from female gDNA and labeled by PCR or nick translation with biotin-16-dUTP (Roche Diagnostics, Mannheim, Germany). The 18S rDNA was obtained from codling moth (*Cydia pomonella*) gDNA using the primers described by Fuková et al. (2005). This probe was labeled by nick translation. For satDNA amplification we designed primers using primer3 (Untergasser et al., 2012) or manually (Supplementary Table 1). PCR was carried out in 20-μl reaction volume containing 1.5 μM of each primer, 1 × Ex *Taq* buffer (TaKaRa), 0.5 U Ex *Taq* polymerase (TaKaRa), 200 μM of each nucleotide and either 50–100 ng gDNA. The thermal cycling profile consisted of initial denaturation at 95°C for 5 min, 30 cycles of denaturation at 94°C for 30 s, annealing at variable temperature (see Supplementary Table 1) for 30 s, elongation at 72°C for 1 min and final elongation 72°C for 3 min. After PCR, the fragments of satDNAs were visualized on a 1.5% agarose gel and monomers were extracted from the gel and re-amplified by PCR. The re-amplified products were sequenced by the Sanger method using the service of SEQme (Dobříš, Czechia) to verify their reliability. For satDNAs probe labeling, a second PCR was done with incorporation of biotin-16-dUTP. For FISH with these probes, we followed the protocol described in Cabral-de-Mello and Marec (2021). The probes were detected by Cy3-conjugated streptavidin (Jackson Immuno Res. Labs. Inc., West Grove, PA, United States).

In experiments in which the W-chromosome painting probe and satDNAs were mapped on the same slide, we applied two rounds of FISH. We first performed W-chromosome painting using the CGH protocol (Traut et al., 1999), then FISH with satDNA probe (Cabral-de-Mello and Marec, 2021). In all FISH experiments, chromosomes were counterstained with DAPI (4′,6-diamidino-2-phenylindole, dihydrochloride; Sigma-Aldrich) and slides mounted with antifade based on DABCO (1,4-diazabicyclo(2.2.2)-octane; Sigma-Aldrich).

## Results

### Karyotypes and Chromosomal Location of 18S rDNA

Diploid chromosome numbers were determined by analysis of mitotic metaphase from wing imaginal disks of females and males stained with DAPI. In *C. perspectalis* and *O. nubilalis* we observed 2*n* = 62 chromosomes [Supplementary Figure 1A; cf. Yasukochi et al. (2016) for chromosome number in *O. nubilalis*]. However, in *D. postlineella* we found a reduced number of chromosomes to 2*n* = 42 (Supplementary Figure 1B). We also counted bivalents in pachytene of females or males, confirming the number of chromosomes in all three species (Supplementary Figures 2A–C). In *D. postlineella*, meiotic bivalents often formed clumps at the pachytene stage (Supplementary Figure 2B), probably due to their longer length compared to the other two species. None of the studied species showed large heterochromatin blocks.

A single sex chromatin body, deeply stained with orcein and of regular spherical shape, was observed in each polyploid nucleus of Malpighian tubules from females, indicating the presence of the W chromosome in all three species (Supplementary Figures 3A,C,E). As expected, sex chromatin was absent in the polyploid nuclei of Malphigian tubules from males (Supplementary Figures 3B,D,E). This finding suggests that the three crambid species have a WZ/ZZ sex chromosome system, which was confirmed by further analysis.

**FIGURE 1:**
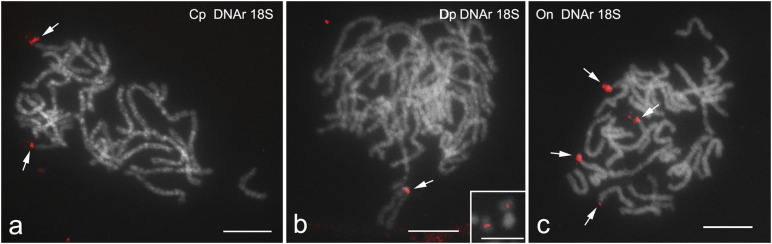
FISH mapping of 18S rDNA clusters in male pachytene bivalents of *Cydalima perspectalis* **(a)**, *Diatraea postlineella* **(b)**, and *Ostrinia nubilalis* **(c)**. Chromosomes were counterstained with DAPI. Arrows indicate hybridization signals of the probes (red). The inset in panel **(b)** shows two homologous mitotic chromosomes from a *D. postlineella* spermatogonium with signals of the 18S rDNA in the interstitial position. Bar = 10 μm.

Fluorescence *in situ* hybridization with 18S rDNA probe applied to male pachytene nuclei revealed significant differences between the species studied in the number and location of rDNA clusters (Figures 1a–c). In *C. perspectalis*, two clusters were found, each located in the terminal position of a different bivalent (Figure 1a), whereas in *D. postlineella* only one interstitial cluster was observed (Figure 1b). In contrast, four terminal clusters in four different bivalents were found in *O. nubilalis* (Figure 1c).

### Differentiation of Sex Chromosomes by CGH and W-Painting Probes

**FIGURE 2:**
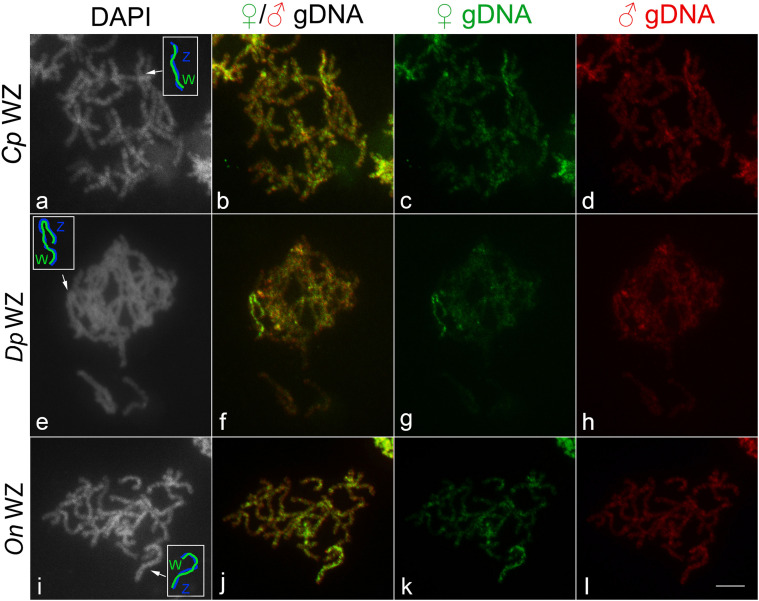
WZ sex chromosome bivalents identified by comparative genomic hybridization (CGH) in female pachytene oocytes of **(a–d)** *Cydalima perspectalis*, **(e–h)** *Diatraea postlineella*, and **(i–l)** *Ostrinia nubilalis*. **(a,e,i)** DAPI staining, **(b,f,j)** merged images of male-derived and female-derived probes, **(c,g,k)** female-derived genomic DNA probe, and **(d,h,l)** male-derived genomic DNA probe. Sex chromosome bivalents are indicated by arrows and schematized in white boxes in panels **(a,e,i)**. Bar = 10 μm.

To investigate the gross molecular differentiation of W and Z chromosomes, we performed CGH. In all three species, the W chromosome showed stronger binding of probes derived from both female and male gDNAs compared to the other chromosomes. In addition, all species showed more intense labeling of female-derived probes in comparison with male-derived probes on the W chromosome, indicating that this chromosome consists mainly of female-specific or female-enriched sequences (Figures 2a–l).

**FIGURE 3:**
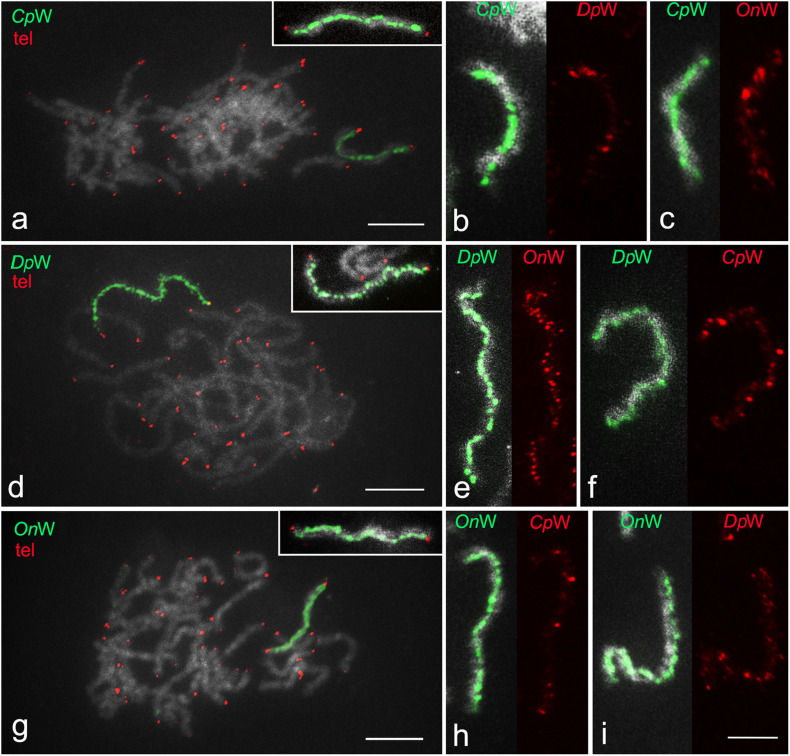
W-chromosome painting in three species of Cambridae. **(a,d,g)** Intraspecies W-chromosome painting (green) and FISH with (TTAGG)*_*n*_* telomeric probe (red). Pachytene oocyte complements are shown in panel **(a)** *Cydalima perspectalis*, **(d)** *Diatraea postlineella*, and **(g)** *Ostrinia nubilalis*. In panels **(b,c,e,f,h,i)**, intraspecies and cross-species paintings of the W chromosome in the WZ bivalent of **(b,c)** *C. perspectalis*, **(e,f)** *D. postlineella*, and **(h,i)** *O. nubilalis* are compared. The probes are indicated directly in the images. Bar = 10 μm.

The W-painting probe specifically highlighted the entire W chromosome at the intraspecies level, allowing easy identification of the sex chromosome bivalent and confirming considerable molecular differentiation of the W chromosome from other chromosomes in all three species. The telomeric probe hybridized only to the end of the chromosomes, which also confirmed that the sex chromosome bivalent is composed of only two elements (WZ) (Figures 3a,d,g). Using cross-species W-painting, we investigated the molecular differentiation of W chromosomes between the three species. Cross-hybridization of W-painting probes resulted in scattered signals on the W chromosome of another species in all cases, but in varying numbers and intensities, depending on the species (Figures 3b,c,e,f,h,i). In general, after FISH with a W-probe from another species, W chromosomes were decorated with several to multiple clusters of hybridization signals along the entire length. The highest intensity of labeling was recorded in the W chromosome of *D. postlineella* using the W-painting probe of *O. nubilalis* (Figure 3e). In contrast, the lowest signal intensity in cross-species W-painting experiments was observed using the W-probe from *C. perspectalis* in the W chromosome of *O. nubilalis* (Figure 3h). Only weak hybridizations signals were observed on some autosomes (results not shown).

### satDNAs Genomic Characterization and Chromosomal Mapping

**FIGURE 4:**
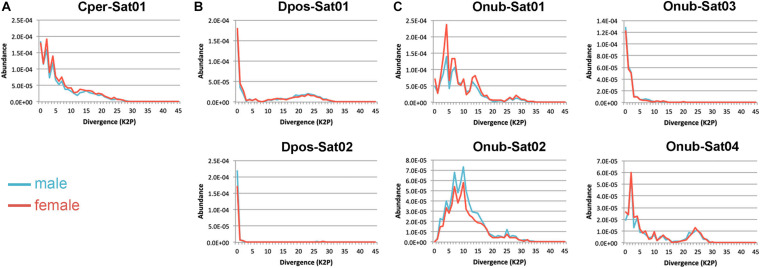
SatDNA landscapes of male (blue) and female (red) repeats for each satDNA identified in **(A)** *Cydalima perspectalis*, **(B)** *Diatraea postlineella*, and **(C)** *Ostrinia nubilalis*.

By clustering analysis, separately for each species, in RepeatExplorer using the TAREAN report, we identified a total of seven putative satDNAs in the three species studied, some of them with high confidence and others with low confidence (a classification provided by TAREAN) (Supplementary Figure 4). One satDNA was identified in *C. perspectalis* (Cper-Sat01), two in *D. postlineella* (Dpos-Sat01 and Dpos-Sat02), and four in *O. nubilalis* (Onub-Sat01, Onub-Sat02, Onub-Sat03, and Onub-Sat04). Subsequent analyses were performed for these seven putative satDNAs, demonstrating their tandem arrangement by dotplot and TRF and also by PCR, which showed a typical ladder-like pattern. Monomers of these satDNAs were highly variable in size, ranging from 123 to 2,244 bp. These satDNAs were A + T-rich with G + C content ranging from 36.44 to 49.11%. They represented only a small portion of the male and female genomes in each species, ranging from 0.01909 to 0.15464%, showing similar abundance between the sexes. The divergences for each satDNA family ranged from 1.32 to 11.34% and were similar between the sexes. These data are summarized in Table 1 and the sequence logos are shown in Supplementary Figure 4. SatDNA landscapes, which were generated to compare the repeats between sexes, aiming to check the possible amplification of variants with distinct degrees of divergence, did not reveal any sex-specific differences (Figure 4). We also searched for all satDNA families at the interspecific level by RepeatMasker and found that they are present in all three genomes, but with a lower proportion compared to the species in which they were identified (Supplementary Table 2). Finally, our Repbase and NCBI searching did not reveal relevant similarity with any described sequences. The sequences were deposited in GenBank under the accession numbers MW369067–MW369073.

**TABLE 1:** Main characteristics of SatDNA families identified in Crambidae genomes.

**Species satDNA family**	**Monomer size (bp)**	**G + C content (%)**	**Abundance (%)**		**Divergence (%)**	
			**Female**	**Male**	**Female**	**Male**
*Cydalima perspectalis*						
Cper-Sat01	2,244	43.36	0.14476	0.12624	7.39	7.14
*Diatraea postlineella*						
Dpos-Sat01	1,391	36.44	0.04980	0.04895	10.1	11.23
Dpos-Sat02	1,380	40.57	0.01929	0.02431	1.32	1.37
*Ostrinia nubilalis*						
Onub-Sat01	528	39.77	0.15464	0.11740	9.38	9.55
Onub-Sat02	453	40.39	0.05552	0.07105	11.34	11.31
Onub-Sat03	957	49.11	0.02807	0.02993	2.05	2.08
Onub-Sat04	123	37.39	0.02839	0.02604	8.86	9.28

**FIGURE 5:**
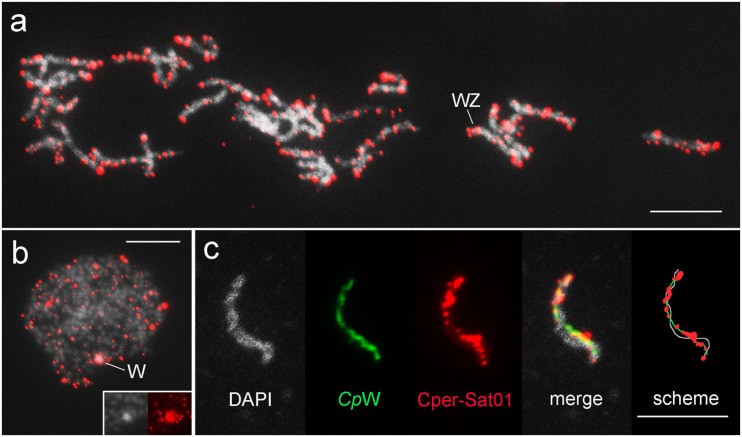
Chromosomal mapping of Cper-Sat01 in pachytene oocyte bivalents **(a,c)** and interphase nucleus **(b)** of *Cydalima perspectalis*. In panel **(c)**, a detail of the WZ bivalent with the W chromosome identified by the W-painting probe (green) is shown, which allows accurate mapping of the distribution of Cper-Sat01 clusters (red). Note the enrichment of the clusters on the W chromosome relative to the Z chromosome. In panel **(b)**, note the strong hybridization signals concentrated on the W chromatin body; the inset shows a detail of the W chromatin body stained with DAPI (gray on the left) and with the probe (red on the right). Bar = 10 μm.

**FIGURE 6:**
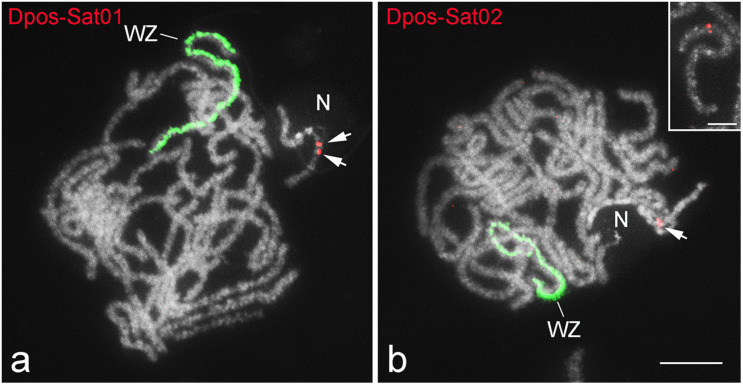
FISH mapping of two satDNAs (red) and W-painting probe (green) in pachytene oocytes of *Diatraea postlineella*. Chromosomes were counterstained with DAPI. Note that both satDNAs show an autosomal location. Dpos-Sat01 **(a)** is located on bivalent carrying the nucleolus (N), while Dpos-Sat02 **(b)** is located on another bivalent. The inset in panel **(b)** shows in detail the interstitial position of Dpos-Sat02. Bar = 10 μm.

**FIGURE 7:**
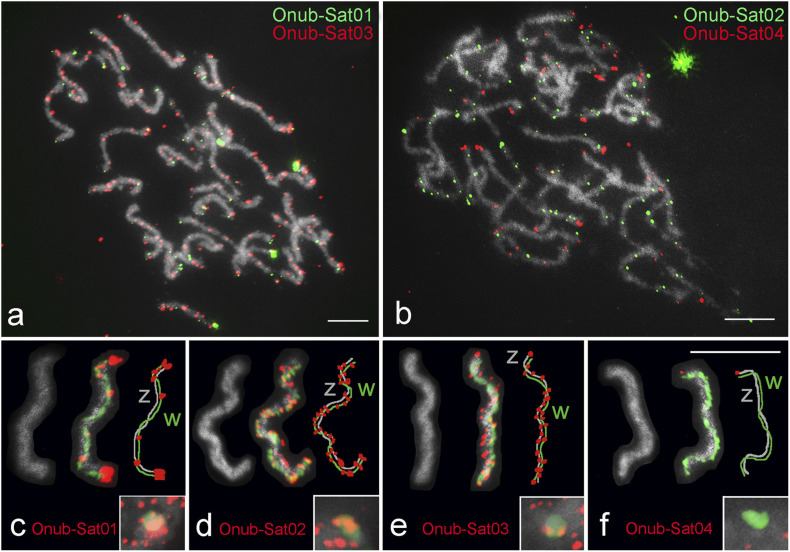
Localization of four satDNAs identified from the genome of *Ostrinia nubilalis*. **(a,b)** Male pachytene. In panels **(c–f)**, selected WZ bivalents from pachytene oocytes stained with DAPI (gray), probed for individual satDNAs (red) and with the W chromosome identified by W-painting probe (green) are shown. Schematic representations of each WZ bivalent showing the W (green) and Z (gray) chromosomes and the satDNA distribution (red) are also given in panels **(c–f)**. The insets in panels **(c–f)** show hybridization signals of the probes on W chromatin from interphase nuclei. Probes are indicated in each image. Bar = 10 μm.

Fluorescence *in situ* hybridization mapping of satDNAs revealed a variable distribution pattern with clusters on autosomes and sex chromosomes (Figures 5–7). Hybridization signals of Cper-Sat01 were scattered in all chromosomes of *C. perspectalis*, with no apparent enrichment in specific chromosomal regions (Figure 5a). However, analysis of interphase nuclei revealed enrichment of this satDNA on W chromatin (Figure 5b). At the pachytene stage, in combined analysis with the W-probe, clusters of Cper-Sat01 were enriched and distributed along the entire length of the W chromosome, but were almost absent on the Z chromosome (Figure 5c). In *D. postlineella*, two clusters of Dpos-Sat01 were located interstitially in a pair of autosomes carrying the nucleolar organizer region (NOR) formed by the major rDNA (Figure 6a). FISH with Dpos-Sat02 probe identified a single cluster that was located on another autosomal pair in the interstitial position (Figure 6b).

Fluorescence *in situ* hybridization mapping of four satDNAs identified in the genome of *O. nubilalis* showed scattered hybridization signals on most chromosomes. However, these satDNAs were distributed differently in various chromosomal regions. Onub-Sat02 and Onub-Sat03 were virtually scattered and formed clusters of small dots along the entire length of most chromosomes, while Onub-Sat01 and Onub-Sat04 were enriched primarily in the terminal regions of the chromosomes (Figures 7a,b). Concerning the WZ sex chromosomes, Onub-Sat01 (Figure 7c), Onub-Sat02 (Figure 7d), and Onub-Sat03 (Figure 7e) showed a similar distribution in both sex chromosomes. Remarkably, large terminal clusters occurred in Onub-Sat01, in addition to several smaller clusters located mainly on the W chromosome (Figure 7c). In contrast, Onub-Sat04 was located exclusively at one termini of the Z chromosome (Figure 7f).

## Discussion

### Karyotypes and Molecular Differentiation of Sex Chromosomes in Crambidae

In this study, we made progress in understanding the basic features of karyotypes and molecular differentiation of sex chromosomes in the economically important family of Lepidoptera, Crambidae. Although the number of Crambidae species karyotyped so far is low, a high variability of haploid numbers, ranging from *n* = 10 to *n* = 41 has been reported (Virkki, 1963; Robinson, 1971; Kageyma and Traut, 2004; Thakur and Gautam, 2013; Yasukochi et al., 2016). These variations in the number of chromosomes below and above the ancestral number of *n* = 31 (Van’t Hof et al., 2013; Ahola et al., 2014) suggest a dynamic karyotype evolution by chromosome fusions and fissions, respectively. Fusions probably also played a major role in reducing the number of chromosomes to *n* = 21 found in this study in *D. postlineella*. The occurrence of large chromosomes in *D. postlineella*, which were difficult to individualize in pachytene compared to *O. nubilalis* and *C. perspectalis* with *n* = 31, also supports the fusion hypothesis. The fact that a related species, the sugarcane borer *D. saccharalis*, also has a reduced chromosome number (*n* = 17; Virkki, 1963), suggests that this is an evolutionary trend in the genus *Diatraea*. In lepidopteran species, a reduction in the chromosome number by fusion is generally more common compared to an increase by fission, which could be attributed to more deleterious effects of fissions in meiotic products (Pringle et al., 2007; de Vos et al., 2020).

The karyotypes of crambid species differed greatly in the number and chromosomal position of rDNA clusters. A single rDNA cluster in the interstitial position, as found in *D. postlineella*, is a common pattern for Lepidoptera (Nguyen et al., 2010). Two terminal rDNA clusters found in the karyotype of C. *perspectalis* were also observed in the Mediterranean flour moth, *Ephestia kuehniella*, from the sister family Pyralidae (Nguyen et al., 2010). Interestingly, the Z-strain of *O. nubilalis* showed four terminal rDNA clusters (this study), while specimens from another population showed five terminal clusters per haploid genome (see Nguyen et al., 2010), suggesting intra-specific variability in the number of rDNA clusters. Besides Crambidae, rDNA variability between species has also been reported in the lepidopteran family Tortricidae, but was mainly manifested by different chromosomal positions of rDNA clusters (Šíchová et al., 2013). The data obtained here for Crambidae add to the growing evidence of high rDNA dynamics in Lepidoptera, which is mainly attributed to ectopic recombination not only in Lepidoptera but also in other insects (Nguyen et al., 2010; Cabral-de-Mello et al., 2011; Ferretti et al., 2019).

We have shown that the studied crambid species have a WZ/ZZ (female/male) sex chromosome system, which is common in Lepidoptera (Sahara et al., 2012). In all three species, the W chromosome is highly molecularly differentiated from the Z chromosome and consists mainly of female-specific and/or female-enriched repetitive sequences, as in some other Lepidoptera (Sahara et al., 2003; Dalíková et al., 2017a,b; Zrzavá et al., 2018). Despite this apparent similarity in gross molecular composition, cross-hybridization experiments revealed that W chromosomes exhibit distinct molecular divergence between species. Nevertheless, some degree of homology has been demonstrated, suggesting partial conservation of repeats on the W chromosomes. However, this degree of homology is not consistent with the phylogeny of Crambidae subfamilies (Regier et al., 2012; Zhu et al., 2018; Léger et al., 2021), since the W probe from *O. nubilalis* (Pyraustinae) showed the highest number of signals in the W chromosome of distant *D. postlineella* (Crambinae), but a lower number of signals in the W chromosome of more closely related *C. perspectalis* (Spilomelinae). These results support the hypothesis of fast independent molecular divergence of W chromosomes with occasional conservation of some repetitive sequences. Partial homology between W chromosomes was also observed in species of the sister family Pyralidae, but the extent of homology was consistent with phylogenetical relationships (Vítková et al., 2007). In contrast, a high molecular divergence was found between the W chromosomes of two congeneric moths of the genus *Abraxas*, Geometridae (Zrzavá et al., 2018).

### satDNAs in Crambidae

The interspecies occurrence of the seven satDNAs identified herein is consistent with the library hypothesis (Fry and Salser, 1977). A common feature of these newly identified satDNAs is the enrichment in A + T base pairs (light satDNAs), similar to the other five satDNAs previously described in Lepidoptera (Lu et al., 1994; Mandrioli et al., 2003; Mahendran et al., 2006; Věchtová et al., 2016; Dalíková et al., 2017b). Interestingly, the monomer length of some of the satDNAs identified herein is high, reaching 2,244 bp for Cper-Sat01 in *C. perspectalis* and more than 1,000 bp for two satDNAs in *D. postlineella*. This is an unusual feature in insects, which generally tend to have satDNA monomer sizes less than 600 bp (Palomeque and Lorite, 2008). Exceptions have been found, for example, in the ant *Monomorium subopacum* with a monomer size of 2.5 kb (Lorite et al., 2004), the kissing bug *Triatoma infestans* with about 1 kb repeat unit (Pita et al., 2017), and in the beetles *Misolampus goutodii* and *Hippodamia variegata* with 1.2 and 2 kb repeat units, respectively (Pons et al., 1993; Mora et al., 2020). It is worth noting that the use of different methods for satDNA prospection could affect the capacity of discovering satDNA families with greater or smaller monomer lengths.

The absence of large heterochromatic blocks in most studied lepidopterans suggests the low abundance of tandem repeats forming long arrays in the chromosomes. This assumption is supported by the extremely low abundance of satDNAs in the three crambid species, in which satDNAs reached a maximum of 0.255% of the genome (mean value between the male and female genomes), as found here in *O. nubilalis*. Such a small proportion of satDNAs is in line with the generally small amount of heterochromatin in Lepidoptera, which is mostly observed only in the W chromosome (Traut et al., 2007) or associated with NOR regions (Nguyen et al., 2010; Šíchová et al., 2013). Only in exceptional cases were numerous heterochromatin blocks observed in other chromosomes (Šíchová et al., 2015, 2016; Zrzavá et al., 2018). This contrasts with other insects, such as hemipterans and grasshoppers, in which a high abundance of satDNAs was revealed by bioinformatics tools (Bardella et al., 2020; Palacios-Gimenez et al., 2020). This may be due to the large genome size of the latter insect groups compared to the generally small size of lepidopteran genomes (Gregory, 2020, Animal Genome Size Database) and also due to predominance of interspersed repeats in Lepidoptera (Talla et al., 2017).

Five of the seven satDNAs identified in Crambidae show a scattered distribution on chromosomes. This appears to be a recurrent pattern in Lepidoptera, as it was also observed in two other satDNAs mapped on chromosomes of moth species from distant families, Saturniidae and Tortricidae (Mahendran et al., 2006; Věchtová et al., 2016). The remaining two mapped satDNAs, MBSAT1 in *M. brassicae* (Noctuidae) and PiSAT1 in *P. interpunctella* (Pyralidae), were limited to sex chromosomes only (Mandrioli et al., 2003; Dalíková et al., 2017b). The predominant scattered distribution is in contrast to patterns observed in organism with large heterochromatin-rich genomes (e.g., Camacho et al., 2015; Pita et al., 2017). This might be caused by the fact that the formation of long arrays of tandem repeats requires a special environment, which is usually lacking in Lepidoptera. Vondrak et al. (2020) recently studied satDNAs of the legume plant *Lathyrus sativus* using ultra-long nanopore reads. They discovered that besides the long arrays of tandem repeats located in centromeric and subtelomeric heterochromatin blocks, satDNAs also formed short arrays in other parts of the genome associated with and probably originated from LTR transposons. The authors speculated that the formation of a long array of satDNA requires restriction of recombination, a process which might reduce the length of the array *via* homologous recombination. This condition is fulfilled, for example, in centromeres and sex chromosomes (Navrátilová et al., 2008; Vondrak et al., 2020). In holokinetic chromosomes of Lepidoptera, which lack a primary constriction (i.e., centromere), there are usually no suitable regions for long arrays of tandem repeats other than the W chromosome, which might be the reason why satDNAs often show a scattered distribution of small clusters. The occurrence of satDNAs in euchromatin, as found in Lepidoptera, has been reported in some insects (Brajković et al., 2012; Palacios-Gimenez et al., 2020; Sproul et al., 2020). Such satDNAs could be located near genes and modulate their expression (Brajković et al., 2012; Feliciello et al., 2015). This aspect deserves more research in Lepidoptera, due to the frequent location of satDNAs in euchromatin and transcription of some satDNAs (Věchtová et al., 2016; Dalíková et al., 2017b).

### Distribution of satDNAs on Sex Chromosomes

Although it is well documented that the lepidopteran W chromosome is mostly composed of heterochromatin rich in repetitive DNA sequences, especially TEs, its specific molecular composition is largely unknown (Abe et al., 2005; Traut et al., 2007, 2013; Sahara et al., 2012). Our study contributed to this knowledge and demonstrated the occurrence of satDNAs on the W chromosomes of *C. perspectalis* and *O. nubilalis*, but not in *D. postlineella*. It should be noted that, unlike Lepidoptera, satDNAs are widely reported in the Y chromosomes of various animals (Giovannotti et al., 2018; Escudeiro et al., 2019; Utsunomia et al., 2019; Ferretti et al., 2020) and plants (Hobza et al., 2015, 2017). Among the tandem sequences identified on the lepidopteran W chromosomes are rDNA in few species (Yoshido et al., 2005; Van’t Hof et al., 2008; Šíchová et al., 2013; Zrzavá et al., 2018) and two satDNAs, PiSAT1 in *P. interpunctella* (Dalíková et al., 2017b) and MBSAT1 in a *M. brassicae* cell line (Mandrioli et al., 2003). However, these satDNAs form conspicuous blocks on the W chromosomes, differing from the satDNAs of *C. perspectalis* and *O. nubilalis*, which are arranged as scattered clusters on the W chromosomes, similar to autosomes. Interestingly, AT-1 of *C. pomonella* is underrepresented on the W chromosome (Věchtová et al., 2016) and two satDNAs identified in *D. postlineella* are located exclusively on autosomes (this study). This demonstrates the great plasticity of satDNA arrangement in lepidopteran genomes, from absence to abundance on the W chromosomes and from a single cluster to a high number of scattered clusters.

The results of our bioinformatic analysis of satDNAs in both sexes of the crambid species studied suggest that in female genomes, ultimately in the W chromosome, there was no extensive amplification of these repeats, as is often reported in the Y or W chromosome in other animals (Palacios-Gimenez et al., 2017; Utsunomia et al., 2019; Ferretti et al., 2020). However, our complementary chromosomal analysis revealed enrichment of some satDNAs on the W (*C. perspectalis* and *O. nubilalis*) or Z chromosome (*O. nubilalis*). Because satDNAs are highly dynamic in copy number (Garrido-Ramos, 2017), the difference in abundance could be attributed to interindividual variability of autosomal clusters, masking the difference in abundance between sex chromosomes.

As observed for euchromatic autosomes, the absence of long arrays surprisingly also applies to the heterochromatic W chromosome in the crambid species studied. Of the seven satDNAs analyzed, four were present on the W chromosome, three of which were abundant there. The patterns of the W-signals of the respective satDNA resembled those observed on the other chromosomes, indicating that the clusters were relatively small and scattered. The only satDNA that formed slightly larger subtelomeric clusters was the Onub-Sat01 (Figure 7c). Of all the lepidopteran satDNAs known today, the only one which forms long arrays in the genome is MBSAT1 in the cabbage moth, *M. brassicae*, which has been shown to be vastly abundant on both the W and Z chromosomes, occupying ca 1.9% of the genome (Mandrioli et al., 2003). However, it would be worth to reanalyze this case, as these results have been obtained from a cell line that showed a drastically reduced chromosome number (*n* = 11) compared to the number reported for the wild population of this species (*n* = 31) (Saitoh, 1959; Sahara et al., 2013). This suggests that the cell line may have undergone genome reconstruction accompanied by amplification of this satDNA, which may not occur in wild populations. Thus, the fact that there are no long arrays of satDNA on the W chromosome that is non-recombining because lepidopteran females lack recombination on all of their chromosomes remains unexplained.

Our CGH results suggest a high abundance of W-enriched and/or W-specific sequences on the W chromosome of all three crambid species. Combining CGH with satDNA mapping helped us to better understand nature of these sequences. In *D. postlineella*, the W-enriched and/or W-specific sequences are not satellites because the only two satDNAs we detected are low-copy autosomal repeats. Hence, the hybridization signals of the female gDNA probe, which highlighted the W chromosome, were generated by other types of sequences, such as mobile elements or microsatellites. In the case of *C. perspectalis*, the only identified Cper-Sat01 is abundant on both autosomes and the W chromosome, while it is underrepresented or absent on the Z chromosome. Thus, it is enriched in females and contributes to the W-highlighting using the female gDNA probe. In *O. nubilalis*, satDNAs mapped to autosomes as well as both sex chromosomes, except Onub-Sat04, which was absent on the W and formed a small cluster at one Z-chromosome end. Hence, the W-enriched and/or W-specific sequences are in fact other repeats than the satDNAs studied.

## Conclusion

Our study significantly contributed to the understanding of karyotype diversification, genome architecture, and sex chromosome evolution in Lepidoptera. The karyotypes of the studied species of the family Crambidae differ in some aspects, namely in the gross architecture (i.e., diploid numbers), probably due to chromosomal fusions, and in fine structures, as evidenced by variability in the distribution of repetitive DNAs. We revealed low abundance and high variability of satDNAs in these Crambidae species, which also contributed to the plasticity of sex chromosomes. The W chromosomes are highly differentiated between the three species due to independent evolution, although some degree of random conservation (not consistent with species phylogeny) of some anonymous repeats has been found. Finally, the combination of genomic and chromosomal data allowed the number of satDNAs identified in Lepidoptera to be doubled, opening new avenues for the study of this genome fraction.

## Data Availability Statement

The datasets presented in this study can be found in online repositories. The names of the repository/repositories and accession number(s) can be found in the article/Supplementary Material.

## Author Contributions

FM and DC-D-M contributed to conception and design of the study. DC-D-M and MZ performed the experiments and analyzed the data. DC-D-M, MZ, and FM interpreted the data and wrote the first draft of the manuscript. SK prepared the W chromosome probes. PR obtained samples of *Diatraea postlineella* and ensured the correct determination of this species. All authors contributed to manuscript revision, read and approved the submitted version.

## Conflict of Interest

The authors declare that the research was conducted in the absence of any commercial or financial relationships that could be construed as a potential conflict of interest. The reviewers DS and PR declared a shared affiliation with several of the authors, DC-D-M and MZ respectively, to the handling editor at the time of review.

## Footnotes

1 http://repeatexplorer.org

2 https://github.com/fjruizruano/ngs-protocols

3 https://github.com/lh3/seqtk

## Supplementary Material

Supplementary Figure 1 Mitotic chromosome spreads obtained from wing imaginal disks of female larvae of **(A)** *Cydalima perspectalis* (prometaphase) and **(B)** *Diatraea postlineella* (early anaphase) and stained with DAPI. Bar = 10 μm.

Supplementary Figure 2 DAPI-stained pachytene bivalents obtained from testes (top images). **(A)** *Cydalima perspectalis* (*n* = 31), **(B)** *Diatraea postlineella* (*n* = 21) and **(C)** *Ostrinia nubilalis* (*n* = 31). A schematic drawing of the same pachytene nucleus is shown at the bottom of each panel; this way of schematic representation was used to determine the number of bivalents. Bar = 10 μm.

Supplementary Figure 3 Orcein-stained polyploid nuclei obtained from Malpighian tubule cells of female **(A,C,E)** and male **(B,D,F)** larvae showing sex chromatin status in **(A,B)** *Cydalima perspectalis*, **(C,D)** *Diatraea postlineella*, and **(E,F)** *Ostrinia nubilalis*. Note the presence of a deeply stained W chromatin body (arrows) in females, while it is absent in males. Bar = 10 μm.

Supplementary Figure 4 Cluster layouts and sequence logos for satDNAs. Graphical layouts were obtained from RepeatExplorer output. Sequence logos were generated from full-length consensus sequences from the most frequent k-mers for each satDNA.

Supplementary Table 1 DNA sequences of primers used for PCR amplification of seven satDNAs and 18S rDNA in Crambidae species. Cper, *Cydalima perspectalis*; Dpos, *Diatraea postlineella*; Onub, *Ostrinia nubilalis*.

Supplementary Table 2 Analysis of abundance of identified satDNAs at the interspecific level in the genomes of Crambidae species.
