# Supplementary figures and images for: The Role of Satellite DNAs in Genome Architecture and Sex Chromosome Evolution in Crambidae Moths
Source: Front Genet. 2021 Mar 30;12:661417. doi: 10.3389/fgene.2021.661417 (PMC8042265; doi:10.3389/fgene.2021.661417)

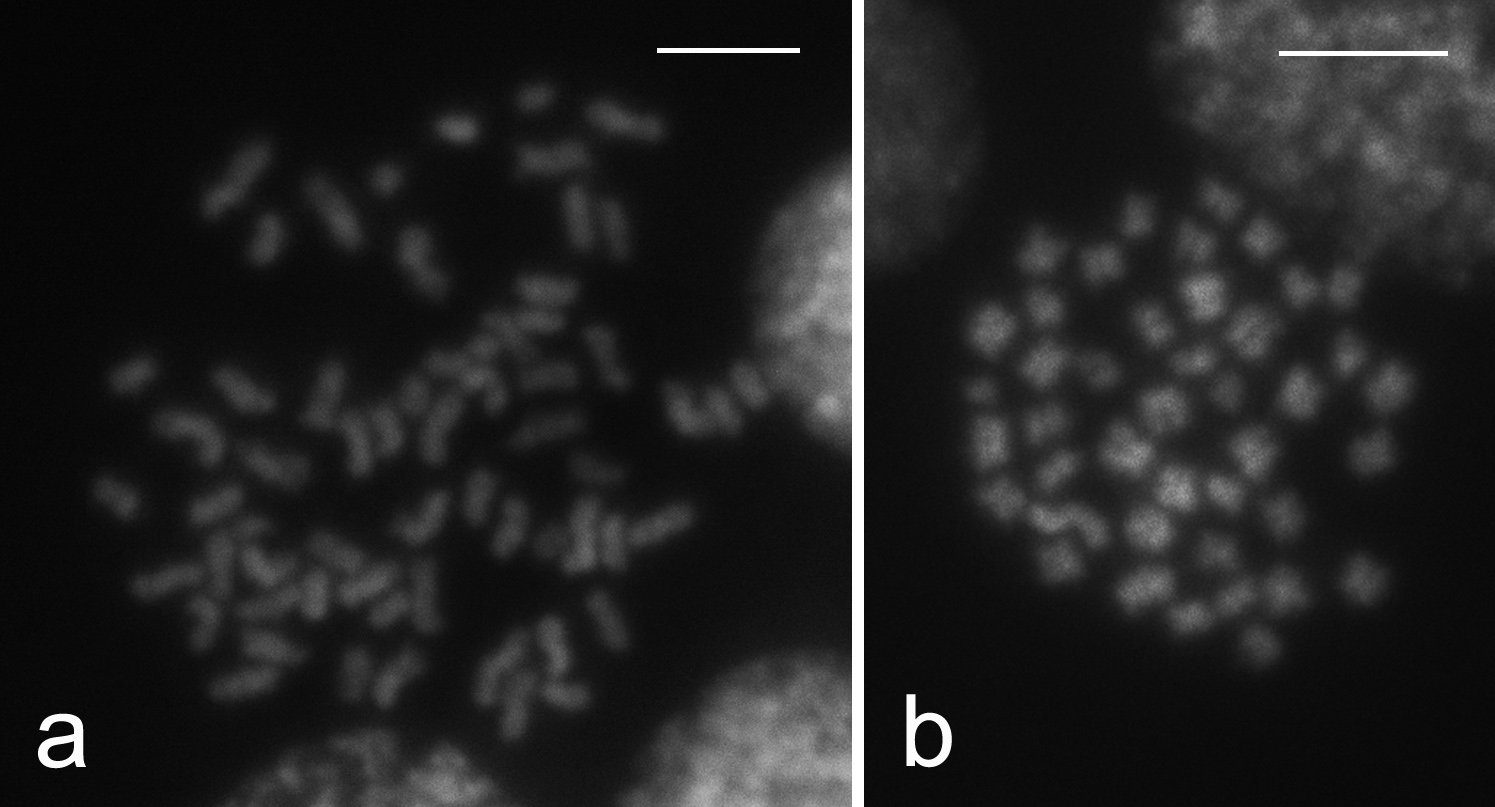

Supplement: Supplementary Figure 1 — Mitotic chromosome spreads obtained from wing imaginal disks of female larvae of (A) Cydalima perspectalis (prometaphase) and (B) Diatraea postlineella (early anaphase) and stained with DAPI. Bar = 10 μm. [file Image_1.JPEG]

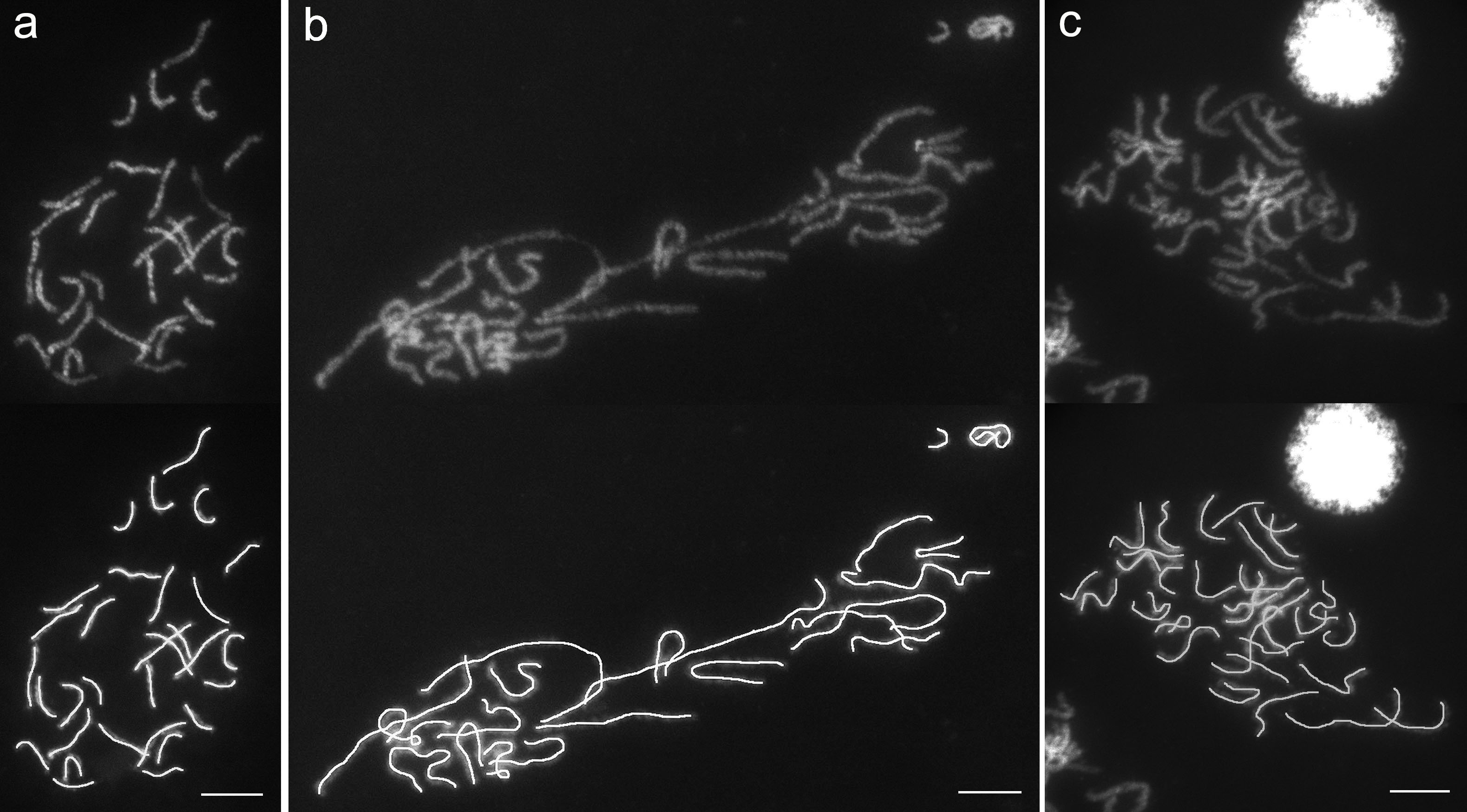

Supplement: Supplementary Figure 2 — DAPI-stained pachytene bivalents obtained from testes (top images). (A) Cydalima perspectalis (n = 31), (B) Diatraea postlineella (n = 21) and (C) Ostrinia nubilalis (n = 31). A schematic drawing of the same pachytene nucleus is shown at the bottom of each panel; this way of schematic representation was used to determine the number of bivalents. Bar = 10 μm. [file Image_2.JPEG]

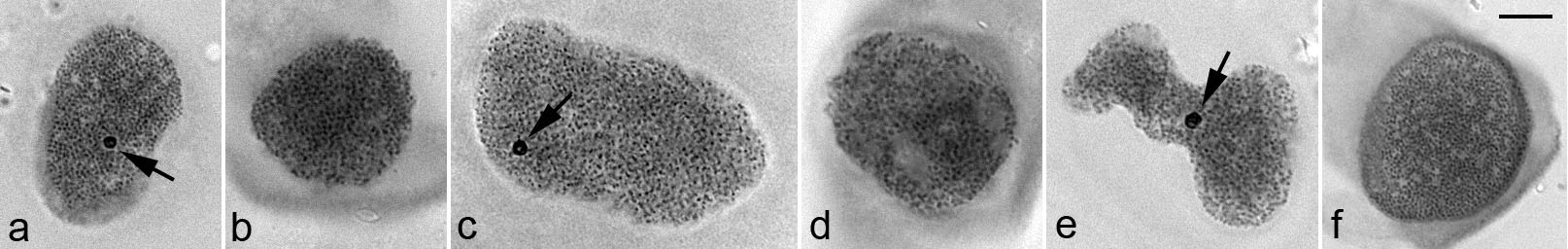

Supplement: Supplementary Figure 3 — Orcein-stained polyploid nuclei obtained from Malpighian tubule cells of female (A,C,E) and male (B,D,F) larvae showing sex chromatin status in (A,B) Cydalima perspectalis, (C,D) Diatraea postlineella, and (E,F) Ostrinia nubilalis. Note the presence of a deeply stained W chromatin body (arrows) in females, while it is absent in males. Bar = 10 μm. [file Image_3.JPEG]
