# Supplementary material for: The Role of Satellite DNAs in Genome Architecture and Sex Chromosome Evolution in Crambidae Moths
Source: Front Genet. 2021 Mar 30;12:661417. doi: 10.3389/fgene.2021.661417 (PMC8042265; doi:10.3389/fgene.2021.661417)
Supplement: Supplementary Figure 4 — Cluster layouts and sequence logos for satDNAs. Graphical layouts were obtained from RepeatExplorer output. Sequence logos were generated from full-length consensus sequences from the most frequent k-mers for each satDNA. [file Data_Sheet_1.PDF]

Cydalima perspectalis (Cper-Sat01)

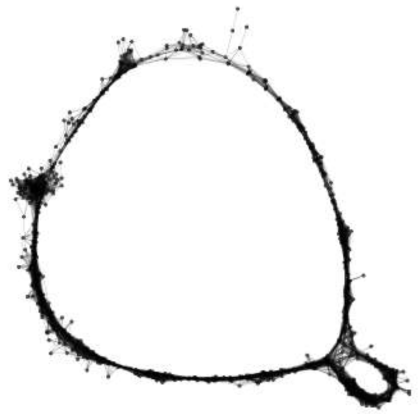

RepeatExplorer graphical layout

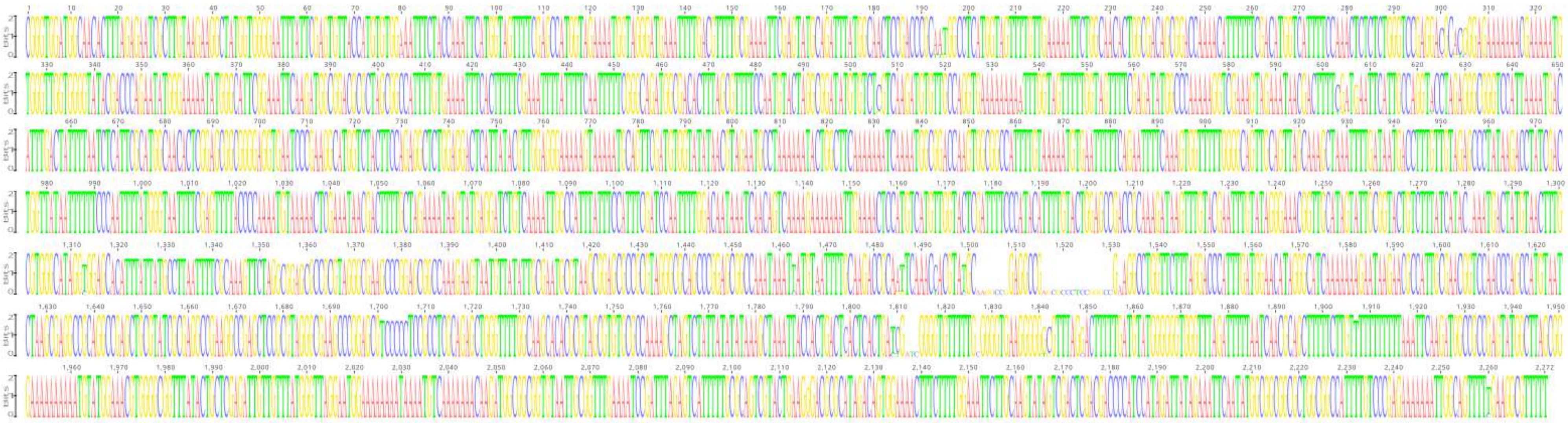

Sequence logo

Diatraea postineella (Dpos-Sat01)

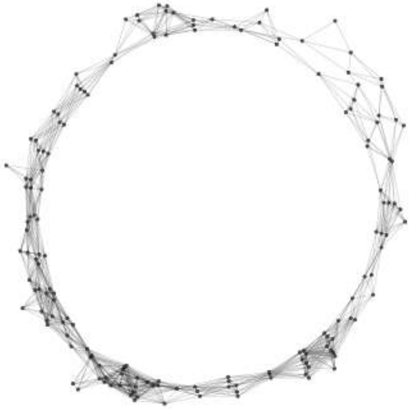

RepeatExplorer graphical layout

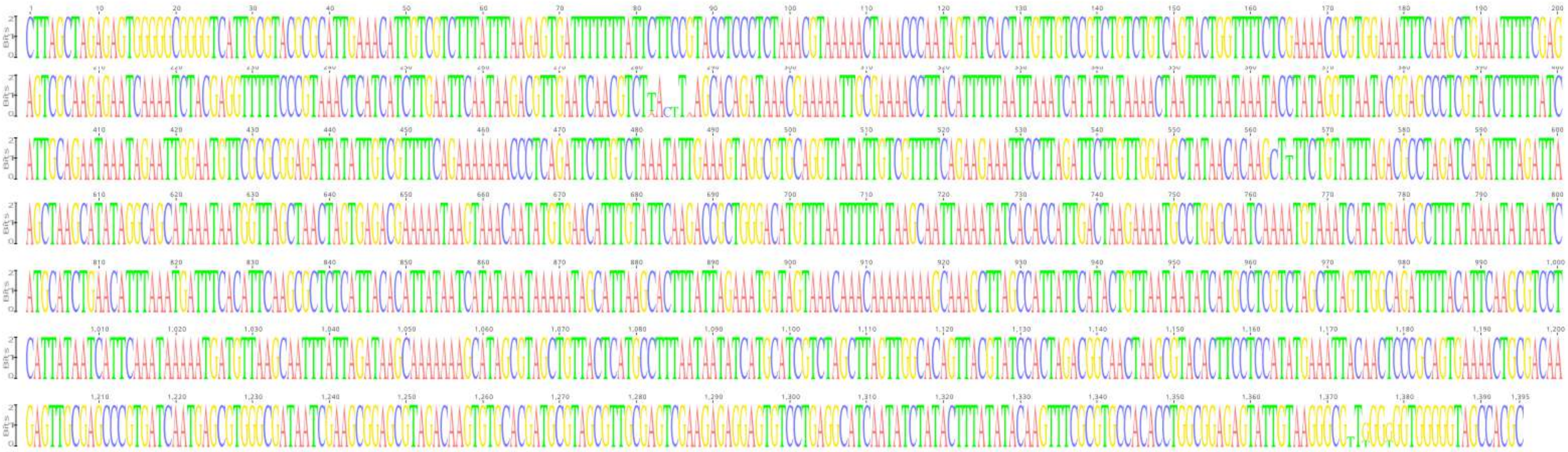

Sequence logo

Diatraea postineella (Dpos-Sat02)

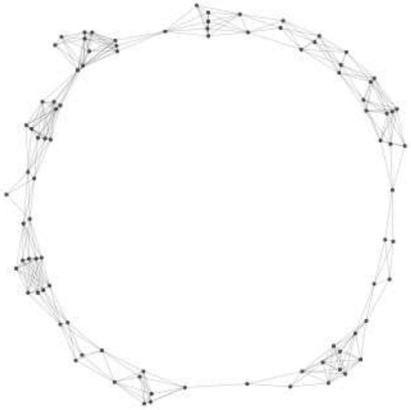

RepeatExplorer graphical layout

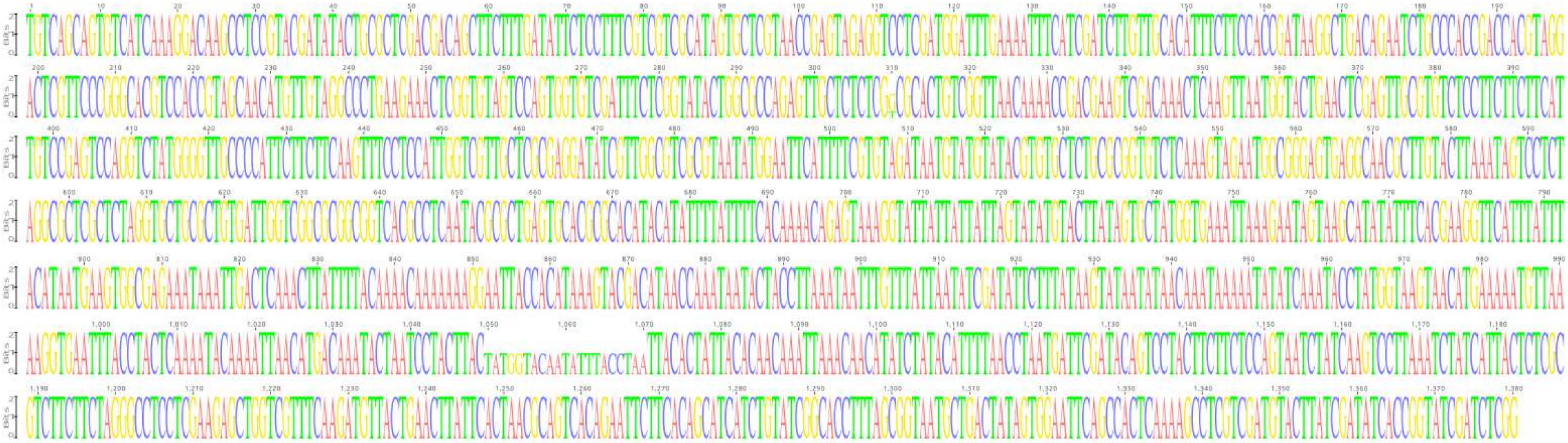

Sequence logo

Ostrinia nubilalis (Onub-Sat01)

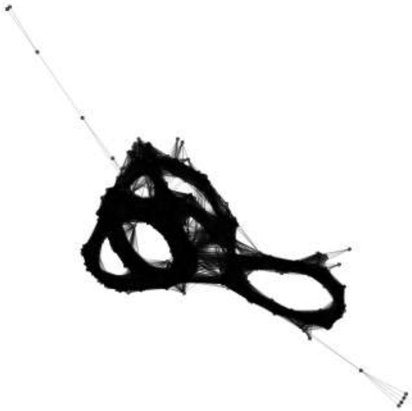

RepeatExplorer graphical layout

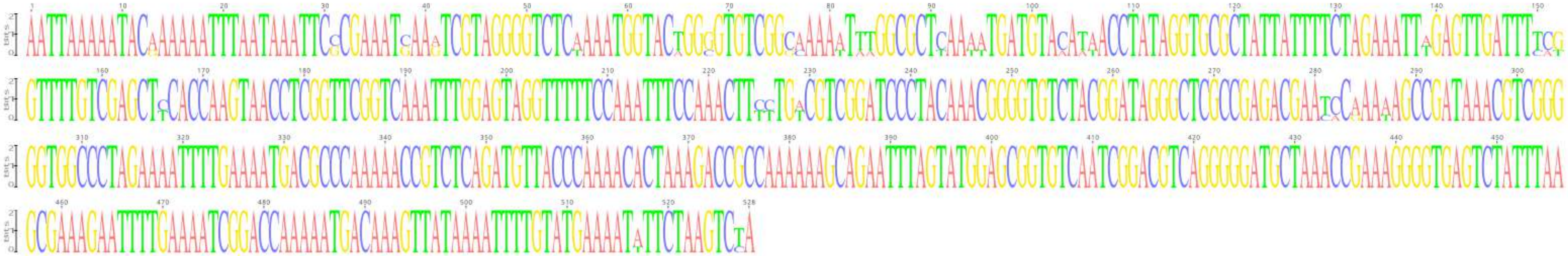

Sequence logo

Ostrinia nubilalis (Onub-Sat02)

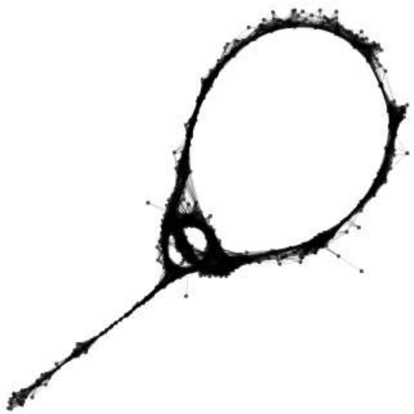

RepeatExplorer graphical layout

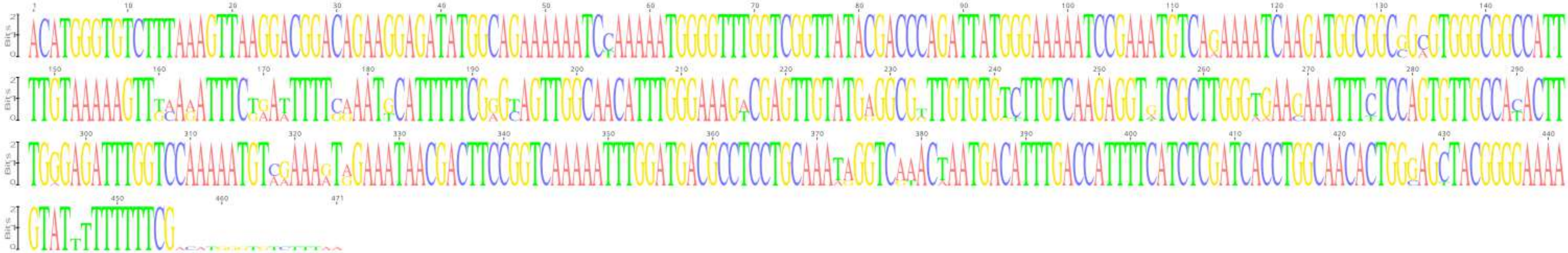

Sequence logo

Ostrinia nubilalis (Onub-Sat03)

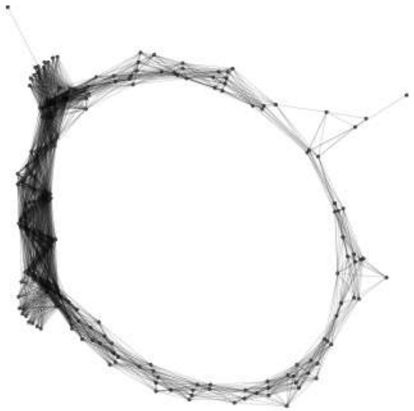

RepeatExplorer graphical layout

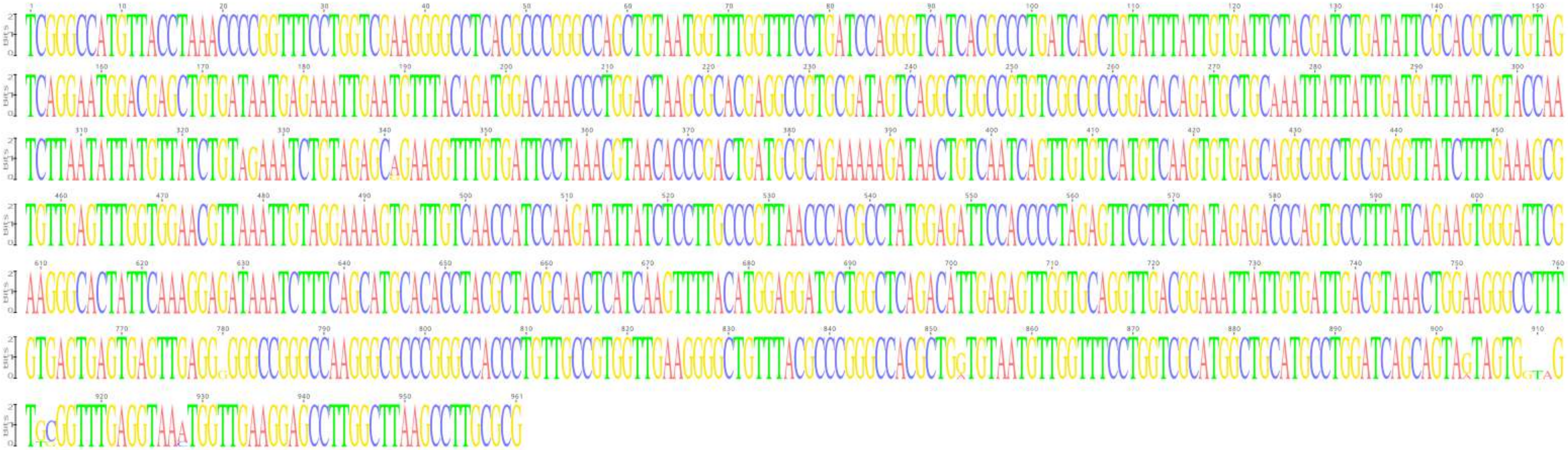

Sequence logo

Ostrinia nubilalis (Onub-Sat04)

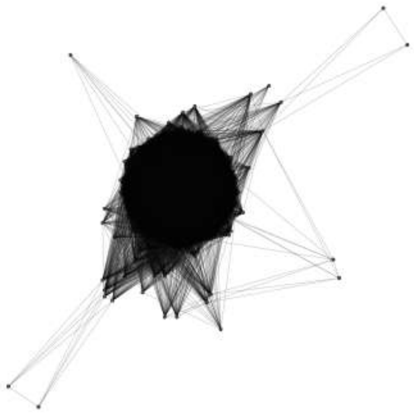

RepeatExplorer graphical layout

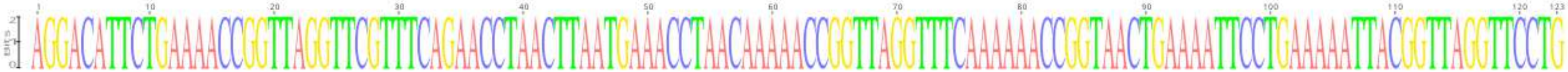

Sequence logo
