# Supplementary material for: The Role of Satellite DNAs in Genome Architecture and Sex Chromosome Evolution in Crambidae Moths
Source: Front Genet. 2021 Mar 30;12:661417. doi: 10.3389/fgene.2021.661417 (PMC8042265; doi:10.3389/fgene.2021.661417)
Supplement: Supplementary Table 1 — DNA sequences of primers used for PCR amplification of seven satDNAs and 18S rDNA in Crambidae species. Cper, Cydalima perspectalis; Dpos, Diatraea postlineella; Onub, Ostrinia nubilalis. [file Table_1.DOCX]

**Supplementary Table S1.** DNA sequences of primers used in PCR experiments of seven satDNAs and 18S rDNA in Crambidae species.

Cper, *Cydalima perspectalis*; Dpos, *Diatraea postlineella*; Onub, *Ostrinia nubilalis*.

| **Sequence** | **Primer F** | **Primer R** | **PCR product size (bp)** | **Annealing temperature** |
| --- | --- | --- | --- | --- |
| 18S rDNA | CGATACCGCGAATGGCTCAATA | ACAAAGGGCAGGGACGTAATCAAC | 1,650 | 50 ^°^C |
| Cper-Sat01 | GGCAGTTTTCGAAGGCGTTT | AATGTGCTCCACCTATGCCC | 1,900 | 60 ^°^C |
| Dpos-Sat01 | GCGAGTCGAAGAGAGGAGTG | AAACATGTCCCAGCGGTCTT | 800 | 60 ^°^C |
| Dpos-Sat02 | TCCTCCATTGGTCGTTGCTC | GCCCTAGAAGAAGACGCGAG | 760 | 60 ^°^C |
| Onub-Sat01 | AAATGACGCCCAAAAACCGT | CCACCCCCGACGTTTATCG | 500 | 60 ^°^C |
| Onub-Sat02 | GAAAATCAAGATGGCGGCGG | CGTATAACCGACCAAACCCCA | 400 | 60 ^°^C |
| Onub-Sat03 | CACGCCCTGATCAGCTGTAT | CCTCAAACCGCACCACTACT | 800 | 60 ^°^C |
| Onub-Sat04 | ATGAAACCTAACAAAAACCGGTT | TCTGAAACGAACCTAACCGG | 110 | 62 ^°^C |
