# Supplementary material for: The Role of Satellite DNAs in Genome Architecture and Sex Chromosome Evolution in Crambidae Moths
Source: Front Genet. 2021 Mar 30;12:661417. doi: 10.3389/fgene.2021.661417 (PMC8042265; doi:10.3389/fgene.2021.661417)
Supplement: Supplementary Table 2 — Analysis of abundance of identified satDNAs at the interspecific level in the genomes of Crambidae species. [file Table_2.DOCX]

**Supplementary Table S2.** Analysis of abundance of identified satDNAs at interspecific level in genomes of Crambidae species.

| **SatDNA family** | **Abundance (%)** | | | | | |
| --- | --- | --- | --- | --- | --- | --- |
|  | ***Cydalima perspectalis*** | | ***Diatraea postlineella*** | | ***Ostrinia nubilalis*** | |
|  | **male** | **female** | **male** | **female** | **male** | **female** |
| Cper-Sat01 |  |  | 0.00395 | 0.00572 | 0.01643 | 0.01193 |
| Dpos-Sat01 | 0.00065 | 0.00065 |  |  | 0.05021 | 0.05013 |
| Dpos-Sat02 | 0.00006 | 0.00003 |  |  | 0.00004 | 0.00005 |
| Onub-Sat01 | 0.00011 | 0.00021 | 0.00026 | 0.00011 |  |  |
| Onub-Sat02 | 0.00069 | 0.00075 | 0.00171 | 0.00247 |  |  |
| Onub-Sat03 | 0.00008 | 0.00012 | 0.00008 | 0.00004 |  |  |
| Onub-Sat04 | 0.00000 | 0.00004 | 0.00003 | 0.00000 |  |  |
